# Photodynamic Therapy Can Modulate the Nasopharyngeal Carcinoma Microenvironment Infected with the Epstein–Barr Virus: A Systematic Review and Meta-Analysis

**DOI:** 10.3390/biomedicines11051344

**Published:** 2023-05-02

**Authors:** Diógenes Germano Fornel, Túlio Morandin Ferrisse, Analú Barros de Oliveira, Carla Raquel Fontana

**Affiliations:** 1 Department of Clinical Analysis, School of Pharmaceutical Sciences, UNESP-São Paulo State University, Araraquara 14801-902, SP, Brazil; 2 Department of Dental Materials and Prosthodontics, School of Dentistry, UNESP-São Paulo State University, Araraquara 14801-903, SP, Brazil; 3 Department of Orthodontics and Pediatric Dentistry, School of Dentistry, UNESP-São Paulo State University, Araraquara 14801-903, SP, Brazil

**Keywords:** photodynamic therapy, nasopharyngeal carcinoma, Epstein–Barr virus, systematic review, meta-analysis

## Abstract

Nasopharyngeal carcinoma is a malignancy from epithelial cells predominantly associated with the Epstein–Barr virus (EBV) infection, and it is responsible for 140,000 deaths annually. There is a current need to develop new strategies to increase the efficacy of antineoplastic treatment and reduce side effects. Thus, the present study aimed to perform a systematic review and meta-analysis of the ability of photodynamic therapy (PDT) to modulate the tumor microenvironment and PDT efficacy in nasopharyngeal carcinoma treatment. The reviewers conducted all steps in the systematic review. PubMed, Science Direct, Scopus, Scielo, Lilacs, EMBASE, and the Cochrane library databases were searched. The OHAT was used to assess the risk of bias. Meta-analysis was performed with a random-effects model (α = 0.05). Nasopharyngeal carcinoma cells treated with PDT showed that IL-8, IL-1α, IL-1β, LC3BI, LC3BII, MMP2, and MMP9 levels were significantly higher than in groups that did not receive PDT. NF-ĸB, miR BART 1-5p, BART 16, and BART 17-5p levels were significantly lower in the PDT group than in the control group. Apoptosis levels and the viability of nasopharyngeal carcinoma cells (>70%) infected with EBV were effective after PDT. This treatment also increased LMP1 levels (0.28–0.50/*p* < 0.05) compared to the control group. PDT showed promising results for efficacy in killing nasopharyngeal carcinoma cells infected with EBV and modulating the tumor microenvironment. Further preclinical studies should be performed to validate these results.

## 1. Introduction

Head and neck cancer comprises a heterogeneous group of malignancies with distinguished etiological factors [1]. The human microbiome has received attention as a risk factor for head and neck cancer [2]. In this regard, *Streptococcus anginous*, *Streptococcus mitis*, *Streptococcus oral*, *Streptococcus gordonii*, *Capnocytophaga gingivalis*, *Prevotella melaninogenica* and *Porphyromonas gingivalis* are examples of microorganisms related to the oncogenesis of head and neck cancer [2]. Moreover, the human papillomavirus (HPV) and Epstein–Barr virus (EBV) strongly correlate to oro- and nasopharyngeal carcinomas, respectively [2].

The EBV (human herpes virus type 4) belongs to the *Gammaherpesvirinae* family and is responsible for infecting more than 90% of the world’s population [3]. Although EBV causes infectious mononucleosis, it is associated with carcinomas and lymphoma carcinogenesis, resulting in 1% of global cancers. Approximately 140,000 people die each year from EBV-related tumors [4].

Nasopharyngeal carcinoma is a malignancy from epithelial cells that extends across the nasopharynx surface, and EBV is one of its etiological factors [4,5,6]. EBV infection divides the level of differentiation of nasopharyngeal epithelial cells into type II (non-keratinizing) and type III (undifferentiated). Hence, a non-keratinizing tumor is predominantly associated with EBV infection [7]. Nasopharyngeal carcinoma prevails in men, with the highest incidence in North Africa and Southeast Asia, particularly in southern China and eastern Malaysia [4]. Additionally, EBV viral load; history of chronic diseases (ear, nose, or throat); genetic factors; environmental exposure; and the consumption of alcohol, tobacco, salted fish, dairy, and lipids are among the etiological factors of nasal carcinoma [4,8].

The latent membrane protein type 1 (LMP1) is present during the latent phase of EBV and is considered an oncogenic protein. Moreover, LMP1 has a higher number of polymorphisms than other genes [9], and it can induce the production of tumors, possibly due to the functional similarity of the anti-protein with tumor necrosis factor (TNF-α), CD40, and tumor necrosis factor type I (TNF-1) [10]. LMP1 can also up-regulate anti-apoptotic genes, down-regulate metastasis suppressors, and promote angiogenesis, pro-inflammatory cytokine activation, and epithelial cell morphology changes [11].

The conventional treatment for nasopharyngeal carcinoma is radiotherapy, chemotherapy, and surgical resection [8]. However, the overall survival rate of patients affected by this carcinoma is still low, and the bad prognoses have remained independent of treatment [12]. Furthermore, other chemotherapy drugs, such as docetaxel, cisplatin, and fluorouracil associated with chemoradiotherapy, have not improved the five-year overall survival and progression-free survival rates [13]. That highlights the absence of new effective therapeutic options for treating these patients [12]. Additionally, conventional treatment (chemotherapy and radiotherapy) is associated with different adverse events, such as skin hyperpigmentation, fatigue, nausea, leukopenia, anemia, hepatotoxicity, and diarrhea [14].

Photodynamic therapy (PDT) involves applying a photosensitizer, followed by a light source in a specific wavelength, to a target tissue [6,15]. After the photosensitizer is sensitized by irradiation, reactive oxygen species are produced, causing cytotoxicity and indirect destruction of tumor cells due to vascular damage [6]. Therefore, PDT might be a promising approach for treating nasopharyngeal carcinoma. Regarding the high prevalence of EBV infection associated with the development of nasopharyngeal carcinoma, the present study aimed to perform a systematic review and meta-analysis of the ability of PDT to modulate the tumor microenvironment and PDT effectiveness in killing nasopharyngeal carcinoma cells infected with EBV.

## 2. Materials and Methods

### 2.1. Protocol and Registration

The present systematic review and meta-analysis were performed according to the Preferred Reporting Items for Systematic Reviews (PRISMA) statement [16]. The study was registered in the Open Science Framework (OSF) (registration DOI: 10.17605/OSF.IO/ACUVG).

### 2.2. Data Extraction and Study Question

The research question was based on the PICO strategy for systematic exploratory reviews [16], where P = nasopharyngeal carcinoma cells infected with EBV; I = photodynamic therapy (PDT); C = PDT associated with another therapy, the absence or application of another treatment instead of PDT, or nasopharyngeal carcinoma cells not infected with EBV; O = the primary outcome was chemokine and interleukin levels and the second one was the viability of nasopharyngeal carcinoma cells infected with EBV and LMP1 levels. The present study aimed to answer the following focused questions: What is the efficacy of PDT in reducing nasopharyngeal carcinoma cells infected with EBV? Moreover, can PDT modulate the inflammatory microenvironment in this tumor infected with EBV?

### 2.3. Eligibility Criteria

The inclusion criteria for the systematic review were in vitro studies that used PDT to treat nasopharyngeal carcinoma cells infected with EBV and cell lines from humans. There was no restriction on types of language, photosensitizer, and cell line. The exclusion criteria were observational studies and clinical trials in humans; book chapters; letters to the editor; conference abstracts; theses; dissertations; case reports; and studies with nasopharyngeal carcinoma cells not infected with EBV, without evaluating the photoinactivation of EBV, and with cell lines from animals.

### 2.4. Search Strategy

Two independent examiners were calibrated in a previous pilot study to perform the steps for article selection. The electronic search was performed in PubMed, Science Direct, Scopus, Scielo, Lilacs, EMBASE, and the Cochrane library databases. The search words were (((Epstein-Barr) OR (Epstein-Barr virus)) OR (EBV)) AND (Photodynamic therapy). The Kappa calibration (0.87/*p* < 0.01) between the examiners was an “almost perfect” agreement. Mendeley Reference Software was used to detect and eliminate duplicates. After the eligibility step, the data were extracted from the selected articles, analyzed, and discussed. Any disagreement during the process was solved before proceeding to the next steps by reaching a consensus. The following data were extracted from the included studies: first name of the author, year of publication, study design, cell lineage, sample size, evaluated group, photosensitizer, wavelength (nanometers), irradiation time (minutes), incubation time of the photosensitizer, light dose, and main results.

### 2.5. Risk of Bias Assessment

This step used the OHAT Rob Rating tool adapted for in vitro studies [17,18]. There were four answer alternatives for each question: (i) definitely low (++) there is direct evidence to affirm the answer to the question; (ii) (+) there is indirect evidence to affirm the answer to the question; (iii) (−) there is indirect evidence to respond negatively to the question; (iv) (−−) there is direct evidence to respond negatively to the question. The question “Were there no other potential threats to internal validity?” referred to a bias related to statistical approaches (sample size calculation, normality and homoscedasticity evaluations, and inferential text details) [19].

### 2.6. Meta-Analysis

The meta-analysis used the random-effects model, the standard mean difference in effect measurement. A forest plot was made to evaluate the results better. The trim-and-fill method was used to detect publication and meta-analysis biases. Heterogenicity levels above 50% were considered high (I^2^ > 50%). R software, version 3.6.3, and Rstudio with the “META” package were used to conduct quantitative approaches (α = 0.05) and build the graphs.

## 3. Results

### 3.1. Search Results

**Figure 1:**
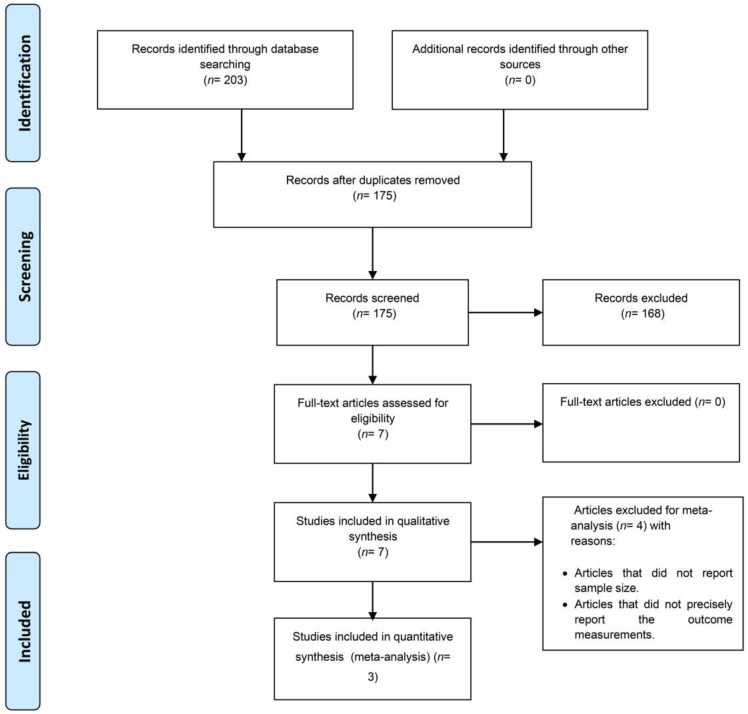
Flowchart summarizing the search results of the present study.

The flowchart in Figure 1 summarizes the article selection process. The electronic search yielded 203 articles. Accordingly, 175 articles remained for selection. After title and abstract screenings, 168 articles were excluded because they did not meet the eligibility criteria. Seven studies were eligible for a full-text evaluation. After the full-text assessment, the same seven articles were included in the qualitative analysis, and three were included in the meta-analysis. Four studies were excluded from the quantitative analysis because they did not report the sample size or precisely report the outcome measurement (Supplementary Materials).

### 3.2. Synthesis of Results

The articles included in the present systematic review and meta-analysis ranged in publication dates from 2002 to 2020 [20,21,22,23,24,25,26] (Table 1). The cell lines most frequently used were CNE-2 (57.15%) and C666-1 (57.15%), followed by HK-1 (42.86%). Numerous photosensitizers were used, but two studies evaluated the same one (FosPeg^®®^) [23,25]. The wavelength varied from 585 to 685 J/cm^2^ and the light dose from 0.25 to 20 J/cm^2^. The photosensitizer incubation time ranged from three to 24 h, and four hours was the most frequently used [20,23,26]. Only one study reported the irradiation time [24].

### 3.3. Risk of Bias Assessment

The primary source of bias in all included articles referred to blinding (were research personnel blind to the study group during the investigation?/was the outcome assessment reliable, including the blinding of evaluators?) and details about statistical approaches (were there no other potential threats to internal validity?) (Table 2).

### 3.4. Meta-Analysis

**Figure 2:**
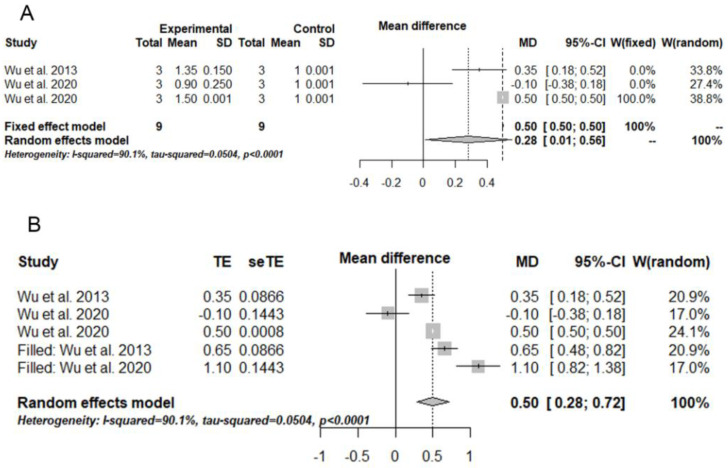
Results of the quantitative approaches used in the present study. (**A**) Meta-analysis illustrated in a forest plot for LMP1 levels with significant results. (**B**) Trim-and-fill results illustrated in a forest plot. This statistical test detected publication and meta-analysis biases, which were corrected. The experimental group included nasopharyngeal carcinoma cells infected with EBV and treated with PDT, and the control group consisted of nasopharyngeal carcinoma cells not infected with EBV and without receiving PDT. MD = mean difference; SD = standard deviation; CI = confidence interval; TE = estimated mean; seTE = estimated standard deviation [23,25,26].

The meta-analysis was only possible for LMP1 levels [23,25,26]. Thus, the experimental group included nasopharyngeal carcinoma cells infected with EBV treated with PDT, and the control group consisted of nasopharyngeal carcinoma cells not infected with EBV and without receiving PDT. PDT increased LMP1 levels (mean difference (MD) = 0.28/95% confidence interval (CI) = 0.01–0.56/I^2^ = 90%) (Figure 2a). After detecting the publication bias with the trim-and-fill method and correlating the meta-analysis, MD was 0.50 [0.28–0.72], but the heterogenicity level remained high (I^2^ = 90%) (Figure 2b).

## 4. Discussion

According to the World Health Organization, there are three pathological subtypes of nasopharyngeal carcinomas: keratinized squamous, non-keratinized, and basaloid squamous [27]. Nonetheless, the non-keratinized subtype represents more than 95% of cases in endemic areas and is predominantly associated with EBV infection [7,27]. This tumor is related to a remarkable geographical distribution. Thus, there are other risk factors for developing nasopharyngeal carcinoma in addition to EBV infection, such as host genetics and environmental aspects (e.g., salted fish consumption) [28,29].

Tumor-derived epithelial cells are susceptible to ionizing radiation, which explains why radiotherapy is the primary treatment modality for non-metastatic nasopharyngeal carcinoma [28]. Chemotherapy combined with radiotherapy is essential and highly indicated for advanced locoregional diseases. Patients with metastatic nasopharyngeal carcinoma are a heterogeneous group, and although chemotherapy is the mainstay treatment modality at this stage, individualized treatment is increasingly required [28,30]. A high dose of radiation or chemotherapy causes acute and later side effects. Oral mucositis, dermatitis, xerostomia, and dysphagia are the main acute toxicities associated with radiotherapy, and xerostomia, sensorineural hearing loss, osteoradionecrosis, trismus, and hormonal dysfunction (e.g., hypothyroidism) are the described later effects [28]. Hematological discrepancies are the main toxicities when administering chemotherapy in nasopharyngeal carcinoma patients [28].

Despite advances in radiotherapy and chemotherapy for treating nasopharyngeal carcinoma, the overall survival rate is still poor and the side effects reduce the quality of life of patients diagnosed with this tumor [12,28,31]. In this scenario, PDT is a treatment option for different cancers [19,32,33,34]. There is a current lack of clinical trials evaluating the effectiveness of PDT for nasopharyngeal carcinoma, which is the main reason for developing this present systematic review on in vitro studies.

Nasopharyngeal carcinoma cells infected with EBV and treated with PDT significantly increased IL-8, IL-1α, and IL-1β levels, resulting in cell death. PDT also increased IL-1α and IL-1β levels in nasopharyngeal carcinoma cells without EBV infection, but at a lower rate than tumors infected with EBV [21]. Moreover, applying PDT only to the tumor did not affect IL-8 levels. In return, apoptosis [21] and cytotoxicity levels from PDT [23,26] were similar in nasopharyngeal carcinoma cells regardless of EBV infection. That is promising because the oxidative damage of PDT can cause different cell deaths and immunological response pathways depending on EBV infection in nasopharyngeal carcinoma cells.

In particular, IL-8 is an inflammatory mediator mainly related to necroptosis [35] that can play a different role in cancer. However, IL-8 can recruit innate immune cells, starting an immunological response against cancer [36]. That is a strength of PDT over other traditional cancer treatments because PDT can initiate immunogenic cell death accompanied by the exposure and release of damage-associated molecular patterns (DAMPs) [37]. In the context of the tumor microenvironment, cancer cells can die by apoptosis, necroptosis, and autophagy, along with inflammatory molecule release that may modulate the immunological response against cancer [37]. Furthermore, the role of IL-8 in PDT differs from radiotherapy, in which IL-8 induces an epithelial–mesenchymal transition [38] and tumor cell repopulation after radiotherapy via RIP1/RIP3/MLKL/JNK/IL-8 pathways [35], leading to a poor prognosis for cancer patients.

IL-1 is a pro-inflammatory cytokine that participates in nasopharyngeal carcinoma development and is recognized as an oncogenic factor for this tumor. High IL-1 levels are normal in nasopharyngeal carcinoma and are stimulated by T cells infiltrated in the tumor and lipopolysaccharides (LPS) [39]. In this scenario, LPS-containing Gram-negative bacteria can stimulate resident macrophages via TLR4 for TNF and IL-1 secretion, inducing cell proliferation and tumorigenesis [40].

Despite the role of IL-1 in nasopharyngeal processes, there is a lack of knowledge about the action of this cytokine in the tumor microenvironment after treatment. The caspase-1/NLRP3/IL-1 pathway regards inflammasome formation and pyroptosis, which stimulate the activation of inflammatory processes and modulation of immune responses [41]. Thus, PDT might induce nasopharyngeal cell death via inflammasome formation, but further studies should be designed to evaluate this point better.

EBV-induced carcinogenesis in nasopharyngeal carcinoma can explain the differences in inflammatory responses of nasopharyngeal carcinoma cells infected with EBV or not after PDT. There are three EBV latent phases, distinguished by viral antigen expression. Type I shows EBNA1 (EBV nuclear antigen 1) expression, type II presents EBNA1 and LMP1, LMP2, and EBERs (EBV-encoded small RNA), and type III includes a high production of EBNA1,2, LMP1, LMP2, and EBRs [42,43].

LMP1 is an oncogenic protein that stimulates the expression of the epidermal growth factor receptor (EGFR), promoting cell growth by activating the MAP kinase pathway [44,45,46]. In epithelial cells, LMP1 inhibits P53-mediated induction of apoptosis and induces lymphocyte sensibilization to TGF-beta, tempering the immune response against cancer cells [47]. In other words, the distinguished carcinogenesis pathways related to different etiological factors (EBV positive and EBV negative) may be considered the reason for different responses obtained after PDT for nasopharyngeal carcinoma.

LMP1 can also activate oncogenic signaling pathways, causing tumor invasion, metastasis, anti-apoptosis ability, and inhibition of squamous cell differentiation [28]. Higher LMP1 levels indicate a poor prognosis for nasopharyngeal carcinoma patients. The meta-analysis showed higher LMP1 levels in nasopharyngeal carcinoma cells treated with PDT than those not treated with PDT. However, as LMP1 function depends on the activation of NF-κB and STAT3 pathways [48] and the group treated with PDT showed lower NF-κB levels, PDT might make LMP1 dysfunctional.

In other words, higher LMP1 levels can represent a direct response to cell death by oxidative damage from PDT; therefore, LMP1 would not run an oncogenic pathway because NF-κB levels decreased. Although our study did not evaluate this finding, we might also hypothesize that, as LMP1 function depends on the NF-κB and STAT3 pathways, PDT might modulate LMP1 function via the STAT3 pathways in persistent cancer cells. However, future studies should assess these very pathways better. It is also essential to balance the benefits between increased LMP1 levels via the NF-κB and STAT3 pathways and PDT efficiency in killing nasopharyngeal carcinoma cells (>70%). The impact of higher LMP1 levels on clinical trials remains to be analyzed.

BART-miRNAs are transcription factors that potentiate tumor growth, cooperate in immune attack escape, and strengthen anti-apoptosis ability [28]. PDT reduced BART-miRNAs levels, indicating that PDT could also positively modulate tumor-mediated factors, possibly improving the prognosis of nasopharyngeal carcinoma patients.

MMP2 and MMP9 are a family of proteolytic enzymes implicated in the invasion and metastasis of numerous cancers because they degrade extracellular matrix components [49]. MMP2 and MMP9 overexpression is associated with higher tumor grades. Concomitantly, MMP2 overexpression is associated with a higher risk of cancer metastasis, and MMP9 overexpression correlates to lymph node metastasis [49,50]. However, MMP2, MMP9, and other proteolytic enzymes in local inflammations from PDT can cooperate in tissue damage by facilitating a reduction in tumor volume [51].

The role of autophagy in cancer development, growth, invasion, and metastasis has recently been highlighted. In this context, microtubule-associated protein 1B light chain 3B (LC3B) is one of the most studied proteins, and its overexpression is associated with a poor prognosis [52]. PDT triggers autophagy in tumor cells by suppressing AKT-mTOR signaling or up-regulating the AMPK pathway [53,54]. Thus, LC3B overexpression indicates that tumor cells underwent autophagy. Related concerns should be mentioned because surviving tumor cells can obtain resistance over PDT by inhibiting autophagy pathways [55].

The effect of PDT on cancer cells is related to apoptosis or necrosis, and autophagy is an intracellular degradation pathway that can participate in pro-survival or pro-death mechanisms. Thus, autophagy often monitors cellular death by PDT as an attempt to survive oxidative damage [55]. Furthermore, autophagy inhibition can decrease anti-apoptotic proteins, promoting survival and tumor adaption against PDT [56]. The precise mechanisms that can unbalance autophagy from running toward pro-death cells are pivotal for improving the clinical outcomes of cancer patients treated with PDT [41]. Thus, autophagy after PDT with different photosensitizers should be further investigated.

The ATP-binding cassette (ABC) transporters are transmembrane proteins that utilize ATP to transport/efflux diverse compounds across cellular membranes [57]. Among these proteins, ABCB1, ABCC1, and ABCG2 can transport numerous chemotherapy drugs outside cells, causing chemoresistance [58]. PDT could not affect ATP protein levels in nasopharyngeal carcinoma cells infected with EBV, which can be a good response because of the absence of tumor resistance by photodamage in this pathway. The present study adapted the OHAT Rob Rating tool to assess the risk of bias for in vitro studies [17,18]. Most included articles showed a higher risk of bias related to blinding. Although blinding is not frequently used for in vitro studies and is highly required in randomized clinical trials, this methodological approach was accepted, considering that effect size estimates may be overrated. Blinding can also eliminate the observation bias [59].

The findings of the present systematic review and meta-analysis should be understood with caution because only seven articles were included in the systematic review and three in the meta-analysis. Moreover, three of the seven articles were published by the same research group, representing a limitation for the present study. Hence, the studies were conducted with different cell lines, such as CNE-2, C666-1, and HK-1. CNE-2 is a poorly differentiated nasopharyngeal carcinoma epithelioid cell line from a primary tumor biopsy in China [60]. The C666-1 cell line represents an undifferentiated nasopharyngeal carcinoma carrying EBV in long-term cultures [61]. The HK-1 originated from a recurrent (after radiotherapy) differentiated nasopharyngeal carcinoma [62]. These differences can represent distinguished molecular signatures that could cause differences in tumor microenvironment responses after PDT and impact PDT efficacy in killing tumor cells.

In summary, PDT can modulate the tumor microenvironment of nasopharyngeal carcinoma cells and is an efficient treatment against these cells when infected with EBV. However, these findings should be investigated in animals and previous preclinical studies.

## 5. Conclusions

PDT is a promising approach as a treatment for nasopharyngeal carcinoma cells infected with EBV because it can modulate the tumor microenvironment. It also showed significant results in killing nasopharyngeal carcinoma cells infected with EBV. Nevertheless, PDT can easily be associated with other treatments for this tumor.

## Figures and Tables

**Table 1:** Data extracted from the articles included in the present systematic review and meta-analysis.

#	Author	Study Design	Cell Line	Sample Size	Evaluated Group	Photosensitizer	Wavelength (nm)	Irradiation Time (minutes)	Photosensitizer Incubation Time	Light Dose	Results
1	Du et al., 2002 [20]	In vitro	HK-1 CNE-2	6	G1: PDT G2: no PDT	Hypericin	585	ND	4 h (HK-1) 6 h (CNE-2)	0.5 J/cm^2^	**IL-8 (pg/mL)** HK-1/ G1: 168.80 ± 7.93 HK-1/G2: 130.80 ± 5.80 CNE-2/ G1:71.15 ± 9.81 CNE-2/G2: 60.09 ± 2.01
2	Koon et al., 2010 [21]	In vitro	HK-1	ND	G1: HK-1 (EBV+) G2: HK-1 (EBV-) G3: control (no PDT + EBV+)	Zn-BC-AM	682	ND	24 h	0.25–1.0 J/cm^2^	**Apoptosis (PI)** HK-1 (EBV+): 80% HK-1 (EBV−): 60% **IL-1α (pg/mL)** HK-1 (EBV+): 6300 ± 250 HK-1 (EBV−): 3301 ± 500 Control/G3: 1350 ± 250 **IL-1β (pg/mL)** HK-1 (EBV+): 92 ± 5 HK-1 (EBV−): 55 ± 5 Control/G3: 18 ± 2 **IL-8 (pg/mL)** HK-1 (EBV+): 15 ± 1 HK-1 (EBV−): 0 ± 0 Control/G3: 430 ± 25
3	Li et al., 2010 [22]	In vitro	c666-1 CNE-2	3	G1: c666-1 (EBV+) G2: CNE-2 (EBV-)	HMME (7(12)-(1-methoxyethyl)-12(7)-(1-hydroxyethyl)-3,8,13,17- tetramethyl-21H,23H-porphin-2,18-dipropionic)	630	ND	3 h	0.6–14.4 J/cm^2^	**Phototoxicity (clonogenic assay)** There were significant and similar results for G1 and G2, particularly when the intracellular uptake of HMME was balanced between the groups.
4	Wu et al., 2013 [23]	In vitro	c666-1 HK-1 CNE-2	3	G1: c666-1 (EBV+) G2: HK-1 (EBV-) G3: CNE-2 (EBV-)	FosPeg	630	ND	4 h	3.0 J/cm^2^	**Cytotoxicity (MTT)** c666-1: 69% HK-1: 77% CNE-2: 84% **LMP1 mRNA expression** c666-1: 8 ± 1.5 (PDT+) c666-1: 1 ± 0.0 (PDT−) **EBV-miR-BART 1-5p** c666-1: 0.75 ± 0.1 (PDT+) c666-1: 1.0 ± 0.0 (PDT−) **EBV-miR-BART 16** c666-1: 0.6 ± 0.25 (PDT+) c666-1: 1.0 ± 0.0 (PDT−) **EBV-miR-BART 17-5p** c666-1: 0.75 ± 0.1 (PDT+) c666-1: 1.0 ± 0.1 (PDT−) **LMP1 protein expression** c666-1: 1.35 ± 0.15 (PDT+) c666-1: 1.0 ± 0.1 (PDT−)
5	Peng et al., 2017 [24]	In vitro	NPC 5-8F NPC 6-10B	ND	G1: PDT G2: PDT + Lovastatin	Photosan II	630	1	24 h	10 J/cm^2^	**Cell viability (Alamar blue)** There were significant results for Lovastatin + PDT for both cell lines.
6	Wu et al., 2020 (a) [25]	In vitro	c666-1	3	G1: 2D culture G2: 3D culture (MCL and MCS)	FosPeg	652	ND	24 h	20 J/cm^2^	**Cell viability (MTT)** 2D: 95 ± 5% MCL: 60 ± 10% MCS: 70% **Apoptosis (Annexin V)** 2D: 30.6 ± 7.7 MCL: 31.0 ± 7.4 MCS: 27.6 ± 7.0 **Necrosis (Annexin V)** 2D: 16.3 ± 8.6 MCL: 9.8 ± 10.6 MCS: 13.5 ± 3.2 **LC3BI protein expression** 2D: 1.5 ± 1.0 MCL: 1.4 ± 1.2 MCS: 0.8 ± 0.5 **LC3BII protein expression** 2D: 1.8 ± 1.0 MCL: 1.25 ± 0.8 MCS: 0.8 ± 0.5 **LMP1 protein expression** 2D: 0.9 ± 0.25 MCL: 1.25 ± 1.0 MCS: 2.0 ± 1.25 **MMP2 protein expression** 2D: 0.7 ± 0.15 MCL: 1.2 ± 0.25 MCS: 1.5 ± 1.0 **MMP9 protein expression** 2D: 0.7 ± 0.15 MCL: 2.2 ± 0.75 MCS: 1.5 ± 0.65 **ABCB1 protein expression** 2D: 0.5 ± 0.25 MCL: 1.5 ± 0.65 MCS: 1 ± 0.8 **ABCC1 protein expression** 2D: 1.0 ± 0.25 MCL: 2.3 ± 1.2 MCS: 1.8 ± 0.1 **ABCG2 protein expression** 2D: 1.7 ± 0.5 MCL: 1.5 ± 0.5 MCS: 1.8 ± 2.0
7	Wu et al., 2020 (b) [26]	In vitro	c666-1 CNE-2	3	G1: c666-1 (EBV+) G2: CNE-2 (EBV-)	H-ALA (5-aminolevulinic acid hexyl ester)	630	ND	4 h	2–4 J/cm^2^	**Cytotoxicity (MTT)** G1: 70% G2: 80% **LMP1 protein expression** G1: 1.5 ± 0.0 Control: 1.0 ± 0 **EGRF protein expression** G1: 0.75 ± 0.16 G2: 0.6 ± 0.0 Control: 1.0 ± 0.0 **p-EGRF protein expression** G1: 0.5 ± 0.3 G2: 0.8 ± 0.16 Control: 1.0 ± 0.0 **NF-ĸB protein expression** G1: 0.8 ± 0.25 G2: 0.8 ± 0.16 Control: 1.0 ± 0.0

EBV: Epstein-Barr virus; NPC: nasopharyngeal carcinoma; PDT: photodynamic therapy. Evaluated groups: G1—group 1, G2—group 2, G3—group 3; PDT: hypericin-based photodynamic therapy; Photosensitizer: hematoporphyrin monomethyl ether. Irradiation time: minutes; ND: not documented; Light dose: J—joules; Results: H-ALA—5-aminolevulinic acid hexyl derivative, MCL—liquid overlay method with agarose base, MCS—hanging drop method, PpIX—protoporphyrin IX. After PDT in nasopharyngeal carcinoma cells infected with EBV, the IL-8 [20], IL-1α, IL-1β [21], LMP1 [23,25,26], LC3BI, LC3BII, MMP2, and MMP9 [25] levels were not higher than control groups. ABCB1, ABCC1, and ABCG2 [25] levels did not show significant results compared to the control group. There were significant results for apoptosis levels [21] and the viability of nasopharyngeal carcinoma cells infected with EBV. However, NF-ĸB protein expression decreased after PDT for the same group compared to nasopharyngeal carcinoma cells not infected with EBV [26]. miR BART 1-5p, BART 16, and BART 17-5p levels also decreased [23].

**Table 2:** Risk of bias analysis according to the OHAT Rob Rating tool adapted to assess the risk of bias of in vitro studies included in the systematic review.

Questions/Studies	Du et al., 2002 [20]	Koon et al., 2010 [21]	Li et al., 2010 [22]	Wu et al., 2013 [23]	Peng et al., 2017 [24]	Wu et al., 2020 (a) [25]	Wu et al., 2020 (b) [26]
Was the administered dose or exposure level adequately randomized?	++	++	++	++	++	++	++
Were study group allocations adequately concealed?	++	++	++	++	++	++	++
Were the experimental conditions identical across study groups?	++	++	++	++	++	++	++
Were research personnel blind to the study group during the investigation?	−	−	−	−	−	−	−
Were outcome data complete without attrition or exclusion from the analysis?	++	++	++	++	++	++	++
Was the exposure characterization reliable?	++	++	++	++	++	++	++
Was the outcome assessment reliable (including the blinding of evaluators)?	−	−	−	−	−	−	−
Were there no other potential threats to internal validity?	−−	−−	−−	−−	−−	−−	−−

++ = direct evidence to affirm the question; − = indirect evidence to respond negatively to the question; −− = direct evidence to respond negatively to the question.

## Data Availability

Not applicable.

## Supplementary Materials

The following supporting information can be downloaded at: https://www.mdpi.com/article/10.3390/biomedicines11051344/s1. The reference [63] is cited in the Supplementary File.
